# Evaluation de la pratique de l’échocardiographie transœsophagienne (ETO): cas du Centre Hospitalier de Dax

**DOI:** 10.11604/pamj.2021.38.296.22464

**Published:** 2021-03-22

**Authors:** Jean Timnou Bekouti, Manitra Rambolarimanana, Akuvi Claude Adossou, Mialy Ravakiniaina Ranaivosoa, Roberto Prudencio, Pierre Bolarin Lawani, Alain Ranjatson, Alpha Diawara, Jean Louis Roynard

**Affiliations:** 1 Service de Cardiologie, Centre Hospitalier de Dax-Côte d’Argent, Dax, 40100, France,; 2 Department of Radiology, Veterans Affairs Hospital, Houston, Texas, 77030, USA,; 3 Service de Médecine Interne, Centre Hospitalier de Dax-Côte d´Argent, Dax, 40100, France,; 4 Service de Neurologie, Centre Hospitalier de Dax-Côte d´Argent, Dax, 40100, France

**Keywords:** Evaluation, échocardiographie transoesophagienne, Centre Hospitalier de DAX, Assessment, transesophageal echocardiography, Dax Hospital Center

## Abstract

Le but de ce travail est d´évaluer la pratique de l´échocardiographie trans-œsophagienne au Centre Hospitalier (CH) de DAX, selon les indications, le profil patient, les résultats retrouvés, la rentabilité de l´examen et la concordance avec l´attente des prescripteurs. Il s´agit d´une étude rétrospective transversale, descriptive à visée analytique menée de Janvier 2016 à Décembre 2018 au laboratoire d´explorations cardiovasculaires du CH de Dax. Sur les 460 examens demandés nous en avons retenu 434 pour l´étude. Les variables étudiées étaient le profil démographique des patients, les indications, les résultats, la rentabilité de l´examen et la concordance avec l´attente des prescripteurs. Sur les 434 examens réalisés, l´âge moyen de la population était de 64,37 ans, avec 64,29% de patients de sexe masculin et un sex-ratio de 1,8. Le bilan d´accident vasculaire cérébral (AVC) (63,59%), la recherche d´endocardite infectieuse (16,12%) et l´évaluation thérapeutique de la prise en charge des thrombus intracavitaires (11,75%) étaient les indications majoritaires. Les prescripteurs étaient majoritairement les neurologues (60,83%). L´examen était normal dans 58,99% des cas. Les résultats pathologiques (40,78%) étaient dominés par les thrombi intra-auriculaires (27,65%), les vidanges auriculaires altérées (9,45%), la présence de foramen oval perméable (5,07%), les anévrysmes du septum inter atrial (2,53%) et les endocardites infectieuses (2,76%). L´examen était rentable dans 40,78% des cas et les résultats étaient concordants à l´attente des prescripteurs dans 39,86%. Notre étude sur l´échocardiographie transœsophagienne a permis de montrer que les indications sont dominées par le bilan d´accident vasculaire cérébral. C´est un examen à faible taux de complications, rentable et avec des résultants concordant avec l´attente des praticiens.

## Abstract (en)

The purpose of this study is to assess the role of transesophageal echocardiography at the Dax Hospital Center based on indications, patients´ profile, results, cost-effectiveness and consistency with prescribers´ expectations. We conducted a retrospective, cross-sectional, descriptive and analytical study at the Cardiovascular Laboratory of the Dax Hospital Center from January 2016 to December 2018. The study included 434 exams out of 460 requested. The variables of interest were: demographic profile of the study population, indications, results, cost-effectiveness and consistency with prescribers´ expectations. The average age of patients undergoing the 434 exams included was 64.37 years, male patients accounted for 64.29%, with a sex ratio of 1.8. Stroke assessment (63.59%), the search for infective endocarditis (16.12%) and the assessment of the treatment of intracavitary thrombi (11.75%) were the main indications. Prescribers were mainly neurologists (60.83%). The examination was normal in 58.99% of cases. The pathological results (40,78%) were dominated by thrombi in the auricle (27.65%), altered auricle emptying (9.45%), the presence of foramen oval permeable (5.07%), interatrial septal aneurysm (2.53%) and infective endocarditis (2.76%). The exam was cost-effective in 40.78% of cases and the results were consistent with prescribers´ expectations in 39.86% of cases. Our study transesophageal echocardiography showed that the indications were dominated by stroke assessment. TEE is a cost-effective diagnostic test with a low rate of complications, and whose results are in line with practitioners' expectations.

## Introduction

L´échocardiographie transœsophagienne (ETO) est un examen de pratique courante en cardiologie [1]. L´idée de mettre un capteur TM sur une sonde œsophagienne revient à L. Frazin en 1976, qui avait obtenu ainsi des images d´une qualité exceptionnelle [2]. L´abord transœsophagien, l´invention des sondes multiplan et récemment l´étude tridimensionnelle en temps réel ont permis une analyse fine et précise des structures anatomiques intracardiaques en complément à l´abord transthoracique [1]. L´examen est pratiqué au sein d´une unité d´exploration fonctionnelle cardiologique au Centre Hospitalier (CH) de Dax. L´équipe est composée de trois cardiologues, trois infirmières qualifiées et les examens sont réalisés sur programmation selon le degré d´urgence. La demande relativement importante et la pluralité des indications nous ont conduits à nous interroger sur l´évaluation de sa pratique au sein de notre établissement, objectif principal de notre étude. Les objectifs spécifiques étaient: de décrire les indications, décrire le profil clinique des patients bénéficiant de l´examen, de déterminer sa rentabilité, d´analyser la cohérence des résultats par rapport aux attentes des prescripteurs, et analyser la corrélation entre les indications et les résultats.

## Méthodes

**Type d´étude:** il s´agit d´une étude rétrospective transversale, descriptive à visée analytique menée de Janvier 2016 à Décembre 2018 au laboratoire d´explorations cardiovasculaires du CH de Dax.

**Population d´étude:** sur les 460 examens demandés nous en avons retenu 434 pour l´étude. Les examens non exploitables ont été exclus.

**Matériels:** les examens ont été réalisés par trois cardiologues opérateurs, assistés d´une infirmière sur Appareil General Electric- Vivid E9 et la sonde d´ETO était une Vivid T9 Transducteur 6 Tc-RS.

**Les variables étudiées:** les médecins prescripteurs, le profil démographique des patients, les indications, les résultats et la rentabilité de l´examen. Les données ont été recueillies sur des fiches d´enquête à travers le logiciel écho dédié de l´hôpital « plusecho ». Les variables qualitatives ont été exprimées en nombre et pourcentage, et les quantitatives en moyenne. Les données ont été traitées par EPI Info 7. Le test de Chi^2^ Pearson a été utilisé pour déterminer l´association entre les variables avec un intervalle de confiance de 95%. A été défini comme examen concordant la présence d´une anomalie suspectée par le prescripteur. A été défini comme examen rentable, l´existence d´une anomalie orientant la prise en charge du patient indépendamment de la suspicion par le médecin prescripteur.

## Résultats

**Figure 1:**
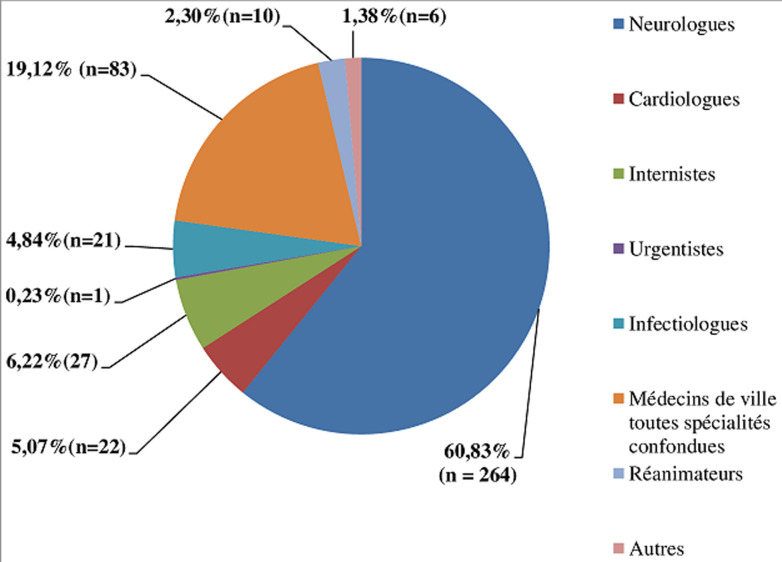
répartition des demandes d’échocardiographie transœsophagienne selon les médecins prescripteurs, analyse de 434 examens au Centre Hospitalier de Dax de 2016 à 2018

**Figure 2:**
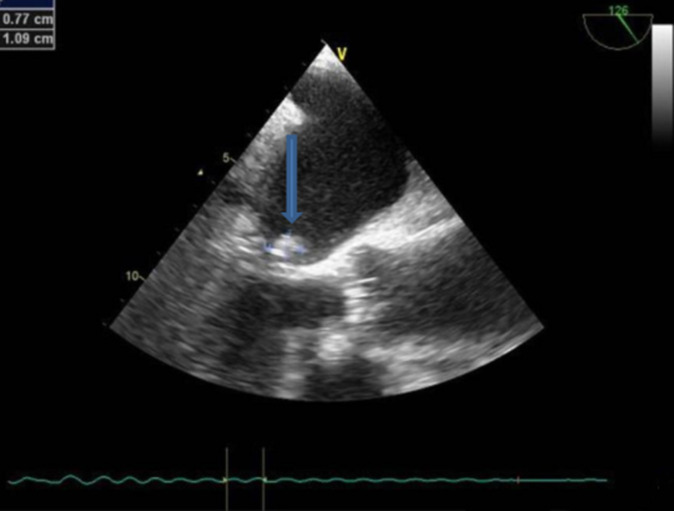
image échocardiographique transœsophagienne d’un thrombus intra-auriculaire gauche chez un patient victime d’accident vasculaire cérébral au en Centre Hospitalier de Dax

Nous avons retenu 434 examens sur 460, soient 94,34%. L´âge moyen était de 64,37 ans (19-92), le sexe masculin était prédominant (64,29%) soit un sexe ratio de 1,8. Aucune complication n´a été rapportée. Les médecins prescripteurs étaient principalement les neurologues (60,83%). Les cardiologues représentaient 5,07% des prescripteurs (Figure 1). Les principales indications étaient représentées par le bilan d´AVC (63,59%), suivies de la recherche d´endocardite infectieuse (16,12%) et le suivi d´un thrombus intracavitaire (11,75%) (Tableau 1). Les résultats étaient rentables dans 40,78% avec comme principales anomalies, les thrombi intra-auriculaires dans 27,65% (Figure 2), les vidanges auriculaires altérées (9,45%), la présence de FOP (5,07%), les ASIA (2,53%), les endocardites infectieuses (2,76%), la présence d´insuffisance mitrale sévère dans 0,92% et d´une bicuspidie aortique dans 0,46% (Tableau 2).

**Tableau 1:** répartition des 434 échocardiographies transœsophagiennes selon les indications au Centre Hospitalier de Dax entre 2016 et 2018

Indications	Effectif n	Pourcentage %
Accident vasculaire cérébral	276	63,59
Recherche d´endocardite infectieuse (EI)	70	16,12
Evaluation thérapeutique d´un thrombus intra-cavitaire	51	11,75
Evaluation d´une prothèse valvulaire	16	3,68
Bilan pré-cardioversion	10	2,30
Bilan diagnostique d´une valvulopathie	5	1,15
Bilan péri-opératoire chez les patients porteurs de valvulopathie	2	0,46
Bilan diagnostique d´une dissection aortique	1	0,23
Autres indications	15	3,46

**Tableau 2:** répartition des 434 échocardiographies transœsophagiennes selon les résultats obtenus au Centre Hospitalier de Dax entre 2016 et 2018

Résultats	Effectifs n	Pourcentage %
Thrombus intra-auriculaire	120	27,65
Vidange auriculaire altérée	41	9,45
Contraste spontané	24	5,53
FOP	22	5,07
Endocardite infectieuse	12	2,76
ASIA	11	2,53
Athérome aortique emboligène	5	1,15
IM sévère	4	0,92
Thrombus intra-cavitaire autre que l´auricule gauche	2	0,46
Bicuspidie	2	0,46
Anévrisme de l´aorte ascendante	2	0,46
CIA	1	0,23
Valve aortique dégénérative	1	0,23
Agénésie auriculaire	1	0,23
Dissection aortique	1	0,23
Autres	4	0,92

**Légende:** FOP = foramen ovale perméable; ASIA = anévrisme du septum inter-atrial; IM = insuffisance mitrale; CIA = communication inter-atriale

Les résultats étaient concordants à l´attente des prescripteurs dans 39, 86%. Dans le cadre du bilan d´AVC, 51,84% des anomalies à l´ETO étaient associées à la survenue d´AVC. Parmi elles, l´existence d´un FOP (p = 0,0002) ou d´un ASIA (p=0,02) ressortaient comme significativement associées à un AVC. La présence de thrombus (p = 0,08), et l´athérome aortique complexe (p=0,25) quant à eux n´étaient pas statistiquement significatifs. Il existait également une forte association entre contraste spontané intra cavitaire et thrombus intra auriculaire (p = 0,009). Il en était de même entre l´altération des vitesses de vidange auriculaire et la présence de thrombus intra-auriculaire (p = 0,02). En ce qui concerne l´endocardite infectieuse, il existait une forte corrélation entre la présence de signes évocateurs chez les médecins prescripteurs et la positivité des ETO (p = 0,0000000001). Nous avons aussi retrouvé 2,3% (10/434) ETO réalisées avant cardioversion électrique dont une sur dix a mis en évidence un thrombus intra-auriculaire gauche contre indiquant de ce fait la cardioversion.

## Discussion

Dans notre étude, nous avons retenu 434 examens sur une période de 36 mois. Ce qui est comparable aux séries de la littérature avec 341 examens sur 16 mois rapporté par Charbonnel *et al*. [3], 103 examens sur 48 mois rapporté par Anzouan-Kacou *et al*. en 2016 [4], 142 sur 28 mois par Yaméogo *et al*. en 2015 [5]. Une série américaine a réalisé 202 séries en 03 mois [6]. Il s´agit donc d´un examen de réalisation courante ne nécessitant pas un plateau technique très particulier. Nous n´avons pas retrouvé de complications. Ceci est conforme au faible taux des complications rapportées dans la littérature [7], Coisne *et al*. sur une étude multicentrique française en 2017 ont rapporté des résultats similaires avec une seule complication sur 1718 examens soit 0,05% [8], montrant qu´il s´agit d´un examen certes semi-invasif mais peu associé à des complications.

L´âge moyen de notre population est de 64,37 ans (19-92). Charbonnel *et al*. en 2014 en France ont rapporté un âge moyen de 70 ans (60-80), [3]. Des séries africaines en 2016 et en 2014 ont rapporté des populations plus jeunes (40,6 ans) [4, 5]. Ceci pourrait être lié au profil de populations africaines dont les séries incluaient la recherche de cardiopathies congénitales chez des populations donc plus jeunes. Dans notre série, nous avons noté une nette prédominance masculine avec un sex-ratio de 1,8 similaire à une série française faite en 2014 [3], et une africaine en 2016 [4].

Nos résultats sont dominés par les thrombus intra-auriculaires gauches suivis de l´altération des vitesses de vidange auriculaire et la présence de contraste spontanée intra-auriculaire gauche (Tableau 2). Une série française en 2014 a retrouvé des ASIA (16,4%) suivi des FOP (15,5%) [3] pour une population d´étude exclusivement victime d´AVC. Nous avons retrouvé 27,65% de thrombi intra-auriculaire gauche dont 25,36% chez les patients qui ont comme indication un AVC (Tableau 3). Des études africaines et européennes ont rapporté beaucoup moins [3, 9, 10]. Cette différence pourrait être liée à la taille de l´échantillon et à la différence par rapport aux indications. Nous avons trouvé une association entre AVC et FOP dans 7,61% des cas (Tableau 3). Ce qui est une proportion plus faible par rapport à la littérature [9, 11]. Dans notre série, il existe une corrélation entre AVC et ASIA dans 3,62% des cas. Ce résultat est similaire à une série européenne en 2014 [12]. Une série américaine quant à elle en a rapporté trois fois plus en 1991 [13]. Un athérome aortique potentiellement emboligène était présent chez 3,63% des patients présentant un AVC (Tableau 3). Charbonnel C *et al*. en 2014 ont trouvé 2,93% [3]; une autre revue française a rapporté 12,6% [14]. Parmi les patients ayant des thrombi intra-auriculaires gauches, la moitié ont un contraste spontané (Tableau 3). Notre résultat est supérieur à celui d´une méta analyse européenne en 2014 qui a rapporté 21,3% [12]. Il s´agit tout de même d´un état qui doit être considéré comme pré-thrombotique notamment chez le patient à haut risque cardio-embolique.

**Tableau 3:** corrélation entre variables étudiées sur les 434 examens retenus durant la période 2016 à 2018 au Centre Hospitalier de Dax

I-Association entre AVC et thrombi intra-auriculaire gauche				
	**Absence de thrombus intra-auriculaire n (%)**	**Présence de thrombus intra-auriculaire n (%)**	**TOTAL n**	**p**
Autres indications	108 (68,35)	50 (31,65)	158	0,08
AVC	206 (74,64)	70 (25,36)	276	
**II-Association entre AVC et FOP**				
	**Absence de FOP**	**Présence de FOP**	**TOTAL n**	**p**
Autres indications	157 (97,37)	1 (0,63)	158	0,0002
AVC	255 (92,39)	21 (7,61)	276	
**III-Association entre AVC et athérome aortique complexe**				
	**Aorte non emboligène**	**Aorte emboligène**	**TOTAL n**	**p**
Absence d´AVC	157 (99,37)	1 (0,63)	158	0,25
Présence d´AVC	272 (96,38)	4 (3,62)	276	
**IV-Association entre thrombi intra-auriculaire gauche et contraste spontané intra-cavitaire**				
	**Absence de thrombus intra-auriculaire**	**Présence de thrombus intra-auriculaire**	**TOTAL n**	**p**
Absence de contraste spontané	302 (73,66)	108 (26,34)	410	0,009
Présence de contraste spontané	12 (50)	12 (50)	24	

Le bilan étiologique d´AVC a été la principale indication, suivie de la recherche d´endocardite infectieuse (Tableau 1). Une série à Libreville en 2008 [15] et une cohorte multicentrique française a rapporté une tendance similaire [8]. Bhatia *et al*. ont par contre rapporté une prédominance sur les bilans avant une cardioversion électrique en Amérique en 2012 [6]. Dans notre étude, les neurologues sont les principaux prescripteurs (Figure 1), Anzouan-Kacou JB *et al*. en 2016 ainsi qu´une série américaine ont rapporté une nette domination par les cardiologues [4, 6]. Dans notre série, 40,78% de nos résultats étaient rentables. Yaméogo NV *et al*. en 2014 ont rapporté cependant 71,8% [5], Charbonnel *et al*. 53,9% d´ETO informatives (source cardiaques ou vasculaires d´embolies) et 8,2% d´ETO ont été considérées comme décisives (ayant un plus du diagnostic posé, modifié l´attitude thérapeutique) [3]. Dans 39,86% les résultats étaient concordants à l´attente des prescripteurs, très fortement marqué dans les sous-groupes recherche d´EI et bilan d´AVC.

## Conclusion

Notre étude a permis d´évaluer la pratique de l´ETO dans notre centre. Il s´agit d’une série à effectif similaire à ceux de la littérature, dont les examens se sont déroulés de façon sécuritaire. La majorité des prescripteurs étaient neurologues et de ce fait le bilan d´AVC ressortait comme étant la principale indication. Environ ¼ des patients ont présenté un thrombus intra-auriculaire gauche. Le FOP et l’ASIA étaient significativement associés aux AVC. Plus d´un tiers des examens étaient rentables et plus de la moitié des examens réalisés dans le bilan d´AVC retrouvaient des anomalies habituellement associées. Plus d´un tiers également des examens étaient concordants à l´attente des prescripteurs.

### Etat des connaissances sur le sujet

- L´ETO est de pratique courante et sécuritaire même chez des patients âgés;
- Les indications sont variables selon l’orientation des centres et pathologies recherchées;
- Une étude antérieure ayant définie les notions d´ETO informatives et décisives.

### Contribution de notre étude à la connaissance

- Indications neurologiques dominantes en centre de cardiologie non spécialisées;
- Evaluation de l´ETO selon les concepts de rentabilité et de concordance.

## References

[1] Cheitlin MD, Armstrong WF, Aurigemma GP, Beller GA, Bierman FZ, Davis JL (2003). ACC/AHA/ASE 2003 Guideline Update for the Clinical Application of Echocardiography: A report of the American College of Cardiology/American Heart Association Task Force on practice guidelines (ACC/AHA/ASEcommittee to update the 1997 guidelines for the clinical application of echocardiography). J Am Soc Echocardiogr.

[2] Chassot PG, Bettex DA (2011). Précis d´anesthésie cardiaque: échocardiographie transœsophagienne 1^e^ partie.

[3] Charbonel C, Fanon L, Georges JL, Colonna G, Stefas L, Isroni C (2014). Interet de l'échocardiographie trans-œsophagienne dans l'optimisation de la strategie thérapeutique après un accident vasculaire cérébral ischémique. Ann Cardio Angeiol.

[4] Anzouan-Kacou JB, Christophe K, Charles-Philippe Z, Djenamba BK, Marie-Paule N, Bénédicte B (2016). L´echographie trans-œsophagienne (ETO) à l´Institut d’Abdjan: indications, résultats et rentabilité diagnostique. Cardiovasc J Afr.

[5] Yaméogo NV, Kagambega LJ, Millogo GR (2015). Transesophageal echocardiography practice in Burkina Faso: Situational analysis and development prospects. Pan Afr Med J.

[6] Bhatia RS, Carne DM, Picard MH (2012). Comparison of the 2007 and 2011 appropriate use criteria for transesophageal echocardiography. J Am Soc Echocardiogr.

[7] Flachskampf F, Badano L, Daniel WG, Feneck R, Fox K, Alalan G (2010). Recommendations for transœsophageal echocardiography: update 2010. Eur J of Echocardiography.

[8] Coisne A, Dreyfus J, Bohbot Y, Pelletier V, Collette E, Cescau A (2018). Transœsophageal echocardiography current practice in France: A multicentre study. Arch Cardiovasc Dis.

[9] Bendriss L, Khatouri A (2012). Les accidents vasculaires cérébraux ischémiques, Fréquence des étiologies cardiovasculaires documentées par un bilan cardiovasculaire approfondi À propos de 110 cas. Ann Cardio Angeiol.

[10] de Bruijn SF, Agema WR, Lammers GJ, Van DW, Wolterbeek R, Holman ER (2006). Transesophageal echocardiography is superior to transthoracic echocardiography in management of patients of any age with transient ischemic attack or stroke. Stroke.

[11] Harloff A, Handke M, Reinhard M, Reinhard M, geibel A, Heltzel (2006). Therapeutic, Therapeutic strategies after examination by transesophageal echocardiography in 503 patients with ischemic stroke. Stroke.

[12] McGrath ER, Paikin JS, Motlagh B, Salehian O, Moira K, Kapral, Martin J O´Donnell (2014). Transesophageal echocardiography in patients with cryptogenic ischemic stroke: a systematic review. Am Heart J.

[13] Pearson AC, Nagelhout D, Castello R, Gomez CR, Labovitz AJ (1991). Atrial septal aneurysm and stroke: a transesophageal echocardiographic study. J Am Coll Cardiol.

[14] Benyounes N, Haddour N, Cohen A (2010). Echocardiographie et sources cardiaques d´embolie. Kardiovaskulare Medizin.

[15] Mipinda JB, Ecke NJE, Allognon C, Kombila P (2016). L´échocardiographie transœsophagienne au Centre Hospitalier de Libreville. Cardiol Trop.

